# Thiazide-associated hyponatremia increases the risk of major adverse cardiovascular events among elderly Taiwanese patients

**DOI:** 10.1186/s12877-023-04583-w

**Published:** 2023-12-15

**Authors:** Hsun Yang, Jane Lu Hsu, Yu-Hung Kuo, Kuan-Fu Liao

**Affiliations:** 1 Division of Nephrology, Department of Internal Medicine, Taichung Tzu Chi Hospital, Buddhist Tzu Chi Medical Foundation, Taichung, Taiwan; 2 Department of Marketing, National Chung Hsing University, Taichung, Taiwan; 3 Department of Medical Research, Taichung Tzu Chi Hospital, Buddhist Tzu Chi Medical Foundation, Taichung, Taiwan; 4 Division of Gastroenterology, Department of Internal Medicine, Taichung Tzu Chi Hospital, Buddhist Tzu Chi Medical Foundation, Taichung, Taiwan; 5 College of Medicine, Tzu Chi University, Hualien, Taiwan; 6 Graduate Institute of Integrated Medicine, China Medical University, Taichung, Taiwan

**Keywords:** Thiazide, Hyponatremia, Elderly, TAH, MACE

## Abstract

**Background:**

Thiazide-associated hyponatremia (TAH) has been supposed to increase the risk of major adverse cardiovascular events (MACE) in the elderly. Therefore, this study aimed to evaluate the association of TAH with the risk of MACE in elderly Taiwanese patients.

**Methods:**

Data from the longitudinal generation tracking database (LGTD 2010) of the Health and Welfare Data Science Center (HWDC) were retrospectively assessed. The TAH study group was defined as using > 30 cumulative daily defined doses (CDDDs) thiazide diuretics within one year before diagnosis of hyponatremia. The control group (1:3 propensity score matching) had no diagnosis of hyponatremia but had used > 30 CDDDs thiazide diuretics within one year. Data on MACE were extracted using International Classification of Diseases codes. Outcomes were assessed using a multivariable Cox proportional hazard model and Kaplan-Meier analysis.

**Results:**

A total of 1155 and 3465 individuals were enrolled in the TAH and the control groups, respectively. The rates of MACE (11.1% vs. 7.3%) and death (22.8% vs.12.2%) were significantly higher in the TAH group than the control group. In the TAH group, the adjusted HRs were 1.29 (CI 1.01 ‒ 1.65) for MACE, 1.39 (CI 1.19 ‒ 1.63) for all-cause death, and 1.61 (CI 0.90 ‒ 2.92) for stroke.

**Conclusion:**

TAH in patients above 65-years-old is associated with a 29% higher risk of MACE, 39% higher risk of all-cause death, and 61% higher risk of stroke. This work suggests that thiazides prescription in elderly patients should be more careful. However, further research is required to confirm our findings.

**Supplementary Information:**

The online version contains supplementary material available at 10.1186/s12877-023-04583-w.

## Introduction

Hypertension is a common chronic disease in the elderly and thiazide diuretics are frequently used as a first-line treatment. Thiazides, a current class of diuretics, competitively antagonize the sodium chloride co-transport protein on the distal convoluted tubules of the kidney to inhibit the reabsorption of sodium chloride and achieve diuresis.

Treatment guidelines often classify hydrochlorothiazide and chlorthalidone as thiazide-type and thiazide-like diuretics, and these drugs are the most commonly used thiazide diuretics in major clinical trials. National guidelines from the US recommended thiazide diuretics as first-line agents for the treatment of hypertension, primarily because of their efficacy to reduce cardiovascular complications in major outcome trials such as the Antihypertensive and Lipid-Lowering Treatment to Prevent Heart Attack Trial (ALLHAT) [1] and Systolic Hypertension in the Elderly Program (SHEP) [2]. Most recent US, Canadian, and European guidelines also recommend thiazide diuretics as first-line drugs for patients with hypertension [3–5]. However, in the UK, thiazide-type diuretics are not approved as a first-line treatment for patients with hypertension [6], and thiazide-like diuretics are recommended instead. Thus, there is a knowledge gap related to the efficacy and side effects of these treatments.

Hyponatremia, the most common electrolyte disorder observed in clinical practice, is often difficult to recognize in the early stages, and is a reported side effect of thiazides. Recent research has noted that the incidence of thiazide-associated hyponatremia (TAH) has increased, and may even be as high as 30% [7]. Although hyponatremia frequently develops rapidly after initiation of thiazide diuretics, many cases of hyponatremia occur after a few months or years of use of thiazide diuretics [8]. A review found that the mean time to TAH after the initiation of therapy was 19 days, and the mean serum sodium level of patients with hyponatremia was 116 mmol/L [9]. While many cases of hyponatremia are asymptomatic or mild, patients with acute onset can develop seizures or coma, and TAH can result in serious morbidity and even mortality in hospitalized patients [10–12].

Other side effects of thiazide diuretics include hypokalemia, hyperuricemia, and hyperglycemia, which are commonly seen in the clinic [13–17]. The use of thiazide diuretics has also been associated with significantly higher risks of major adverse cardiovascular events (MACE), particularly stroke, in patients with type 2 diabetes who received intensive blood pressure control [18].

The risk factors for TAH include age, female sex, and possibly low body mass [19–21]; elderly patients may also represent a high-risk group for the occurrence of hyponatremia. Since age is a strongly associated risk factor for hyponatremia, the decision to initiate thiazide diuretics for elderly patients should be made with caution.

Hyponatremia induced by thiazide diuretics is an important topic in the treatment of hypertension; however, the relationship between thiazide complications and subsequent hypertension treatment goals are rarely explored. The goal of aggressive treatment of hypertension is to reduce the risk of MACE and to ultimately reduce mortality; however, hyponatremia increases the duration of hospital stay and is associated with a higher risk of death in hospitalized patients. Hence, there seems a lack of knowledge on the relationships between TAH and MACE in elderly patients.

The purpose of this study was to examine whether TAH adversely influences the risk of MACE in the elderly in Taiwan. We aimed to fill in this knowledge gap and provide a statistic analysis of the risk of MACE associated with use of thiazide diuretics in the elderly population.

## Materials and methods

### Data source

This study assessed data from the Health and Welfare Data Science Center (HWDC). The National Health Insurance (NHI) program was initiated in 1995; 24.02 million people (nearly 100% of Taiwan’s population) were included in this program at the end of 2019 [22]. The 2000 Longitudinal Generation Tracking Database (LGTD 2010) of two million people was established to provide access to health data for academic research; data are made available to researchers upon request. All patient data including date of birth, sex, diagnostic codes, drug prescriptions, and medical procedures claimed in the Taiwan NHI program are included in the database. We retrieved data on patients in the LGTD 2010 with defined diseases using the International Classification of Diseases codes (Ninth Revision, Clinical Modification; ICD-9-CM and Tenth Revision, Clinical Modification; ICD-10-CM).

### Study population and design

This retrospective cohort study was designed to investigate the risk of MACE in elderly patients with TAH. Data on two million patients between 2005 and 2018 were extracted from the LGTD 2010. We collected data on medicines containing thiazide-type diuretics available in Taiwan from 2004 to 2018 (ATC code see supplement); individuals who ever had a prescription of thiazide-type diuretics were potentially eligible for this study. In some extent anti-hypertension medications conjugated varied dose thiazide were also included (ex. Exforge HCT, Sevikar HCT, Co-Diovan, & Hyzaar, etc.). Moreover, we also retrieved data on prescription of antihypertensive medications, non-steroidal anti-inflammatory drugs (NSAIDs), tricyclic antidepressants (TCAs), and other types of diuretic medications for eligible patients.

Eligible individuals with a diagnostic code of hyponatremia (ICD-9-CM Diagnosis Code 276.1 or ICD-10-CM Diagnosis Code E87.1 Hyposmolality and/or hyponatremia) within the study period (whether as an inpatient, outpatient, or ER) and who had been prescribed > 30 cumulative daily defined doses(CDDDs) thiazide-type diuretics within one year before diagnosis of hyponatremia were allocated to the TAH group. The index date for the TAH group was defined as the day hyponatremia was diagnosed. Individuals without a hyponatremia diagnosis coding but who had been prescribed > 30 CDDDs thiazide-type diuretics in one year were eligible for the control group; 1:3 propensity score matching was conducted to select participants for the control group. The index date in the control group was defined as one year after the date thiazides were first prescribed.

**Fig. 1:**
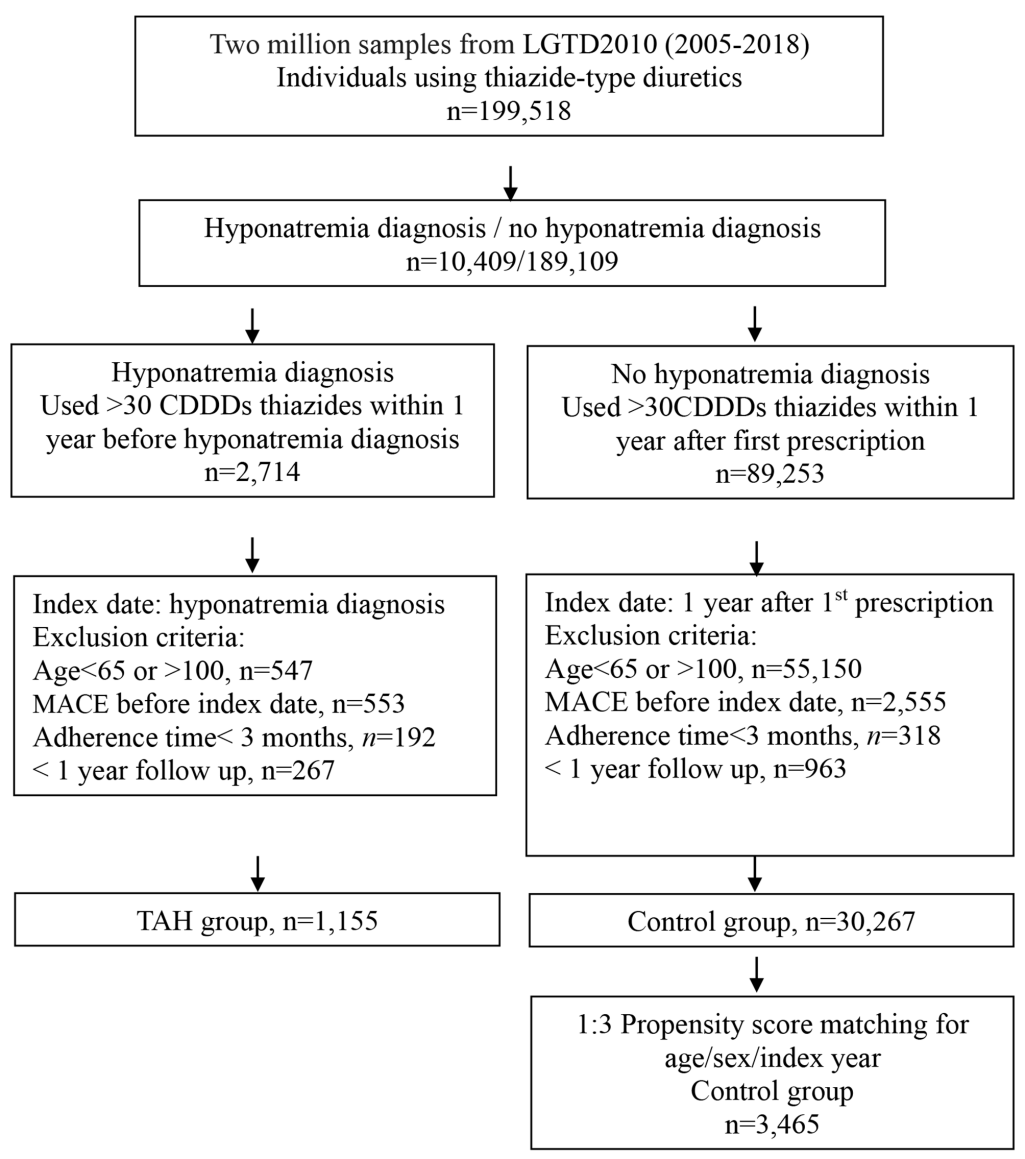
Patient selection flow chart

The exclusion criteria for both the TAH and control groups were age younger than 65-years-old or older than 100-years-old; MACE before the index date; adherence time (index date to event) less than three months; and follow up time less than one year. The patient selection flow chart is shown in Fig. 1.

### End points

A classic composite MACE end-point was used in this study, according to the following corresponding ICD-9-CM & ICD-10-CM codes: (1) Myocardial infarction (MI: ICD-9-CM codes 410–410.9 / ICD-10-CM codes I21, I22); (2) Heart failure (HF: ICD-9-CM codes 428.0–428.10/ICD-10-CM code I50); (3) Cerebrovascular accident (CVA: ICD-9-CM codes 430–437/ICD-10-CM codes I60, I61, I62, I63, I65, I66, I67, I68, G45, G46); (4) Malignant dysrhythmia (MD: IDC-9-CM codes 426.0, 426.12–426.13, 426.51, 426.52, 426.54, 427.1, 427.4, 427.41, 427.42, and 427.5 / ICD-10-CM codes I442, I470, I472, I493, I4901, I4902, I462, I468, I469, I441, I452, I453); and (5) Cardiac shock (CS: ICD-9-CM code 785.51/ICD-10-CM code R570) [23]. MACE after the index date were recorded for both the TAH and control group.

### Statistical analysis

Descriptive analysis was performed for characteristics such as gender, age, disease burden, MACE, and drug use. Descriptive statistics are expressed as the mean and standard deviation (SD) or numbers and percentages. Pearson’s χ^2^ test and the *t*-test were employed to compare the distributions of categorical variables and differences in continuous variables, respectively.

Study endpoints were estimated using a multivariate Cox proportional hazards model; crude hazard ratios with 95% confidence intervals (CIs) are presented. In addition, we adjusted for known risk factors such as age, disease burden, diabetes, and use of other medications; adjusted hazard ratios with 95% confidence intervals were calculated.

The incidence of each type of MACE in the TAH group and the matching control group was expressed in terms of the number of occurrences and the 100 person-years incidence, and the ratios of the incidence of these outcomes between groups were calculated for risk comparison. The crude and adjusted hazard ratios of each MACE outcome, including all-cause mortality, cardiac death, major coronary artery disease, and stroke, were compared. The cumulative rates of MACE in the TAH group and control group were also compared using Kaplan-Meier curves and the log-rank test.

The significance threshold for statistical analysis was set at *P* = 0.05. SAS for Windows (version 9.4, SAS Institute Inc., Cary, NC, USA) and SPSS (version 19.0, SPSS Inc., Chicago, IL, USA) were used for statistical analyses.

## Results

From the two million subjects in the LGTD (2010), we identified 199,518 patients who were prescribed thiazide-type diuretics between 2005 and 2018. Using the diagnostic code for hyponatremia, these patients were classified as having a diagnosis of hyponatremia (*n* = 10,409) or no diagnosis of hyponatremia (*n* = 189,109). Patients diagnosed with hyponatremia who used > 30 CDDDs thiazide diuretics within one year before diagnosis of hyponatremia were eligible for the TAH group (*n* = 2714). Patients without hyponatremia who used > 30 CDDDs thiazide diuretics within one year after first prescription (*n* = 89,253) were eligible for the control group. Patients less than 65-years-old or over 100-years-old, who had a MACE before the index date, adherence (index date to event) of less than three months, or who were followed up for less than one year were excluded. In total, 1155 individuals were enrolled in the TAH group. From the 30,267 eligible individuals, 3465 patients were included in the control group after 1:3 propensity score matching. The patient selection flow chart is presented in Fig. 1. The propensity score matching is meant to show the comorbid disease composition and pattern in each group was similar. The difference in baseline comorbidities between individuals with TAH and controls is very large. Despite propensity score matching, residual confounding from underlying diseases may still be present.

The disease burden presented as CCIS (2.4 ± 1.9 vs. 1.2 ± 1.5; *P* = < 0.001) and proportion of patients with diabetes (43.7% vs. 25.3%; *P* = < 0.001) were higher in the TAH group than the matched control group. The incidence of MACE (11.1% vs. 7.3%) and death events (22.8% vs. 12.2%) were also significantly higher in the TAH group. The differences in the prescription of other medications, including antihypertensive medications, non-steroidal anti-inflammatory drugs (NSAIDs), tricyclic antidepressants (TCAs), and other types of diuretics, are listed in Table 1.

**Table 1:** Baseline characteristics of the TAH group and control group

	TAH group *n* = 1155	Control group *n* = 3465	*P-*value
**Age**	78.9 ± 7.5	78.6 ± 7.4	0.813
**Sex, male**	469(40.6)	1403(40.5)	0.945
**CCIS**	2.4 ± 1.9	1.2 ± 1.5	< 0.001
Myocardial infarction	20(1.7)	28(0.8)	0.007
Congestive heart failure	318(27.5)	544(15.7)	< 0.001
Peripheral vascular disease	61(5.3)	113(3.3)	0.002
Cerebrovascular disease	275(23.8)	371(10.7)	< 0.001
Dementia	109(9.4)	154(4.4)	< 0.001
COPD	260(22.5)	350(10.1)	< 0.001
Connective tissue disease	28(2.4)	31(0.9)	< 0.001
Peptic ulcer disease	276(23.9)	366(10.6)	< 0.001
CKD	172(14.9)	200(5.8)	< 0.001
Hemiplegia	18(1.6)	19(0.6)	< 0.001
Solid tumor	104(9.0)	187(5.4)	
Liver disease	74(6.4)	113(3.3)	< 0.001
**DM**	505(43.7)	878(25.3)	< 0.001
**Event**			< 0.001
None	764(66.2)	2788(80.5)	
MACE	128(11.1)	254(7.3)	
Death	263(22.8)	423(12.2)	
CV death	19(1.6)	20(0.5)	
**Medication**			
ACEI	91(7.9)	276(7.8)	0.925
ARB	819(70.9)	2620(75.6)	0.002
CCB	709(61.4)	1753(50.6)	< 0.001
α-blockers	166(14.4)	304(8.8)	< 0.001
β-blockers	318(27.5)	840(24.2)	0.025
Furosemide	265(22.9)	365(10.5)	< 0.001
Indapamide	38(3.3)	103(3.0)	0.587
Spironolactone	58(5.0)	72(2.1)	< 0.001
Amiloride	52(4.5)	121(3.5)	0.117
NSAIDs	429(37.1)	1094(31.6)	0.001
TCA	59(5.1)	74(2.1)	< 0.001
Diazepam	117(10.1)	182(5.3)	< 0.001
**Follow up (years)**	3.5 ± 1.4	3.8 ± 1.4	< 0.001

*Abbreviation: CCIS, Charlson Comorbidity Index score; COPD, chronic obstructive pulmonary disease;CKD, chronic kidney disease; DM, diabetes mellitus; MACE, major adverse cardiovascular events; ACEI, angiotensin converting enzyme inhibitors; ARB, angiotensin II receptor blocker; CCB, calcium channel blocker; NSAIDs, non-steroidal anti-inflammatory drugs; TCA, tricyclic antidepressant

In the multivariable Cox proportional hazard model (Table 2), TAH was associated with a 56% higher risk of MACE (HR 1.56, CI 1.26‒1.92) in the crude model and 29% higher risk after adjustment (adjusted HR 1.29, CI 1.01‒1.65). However, age was only a weak risk factor, while female sex was associated with a lower risk of MACE. Disease burden represented as Charlson comorbidity index score (CCIS) was a significant risk factor for MACE (crude HR 1.13, CI 1.06–1.20; adjusted HR 1.08, CI 1.01–1.16). However, DM was not associated with the risk of MACE in this model. With respect to medications, only angiotensin II receptor blockers were associated with a lower risk of MACE.

**Table 2:** Multivariable Cox proportional hazard models for risk of MACE

	Crude HR (95%CI)	*P*-value	Adjusted HR (95%CI)	*P*-value
**TAH**	1.56(1.26‒1.92)	< 0.001	1.29(1.01‒1.65)	0.039
**Age**	1.04(1.02‒1.05)	< 0.001	1.03(1.01‒1.05)	< 0.001
**Sex, male**	1.11(0.91‒1.36)	0.311		
**CCIS**	1.13(1.06‒1.20)	< 0.001	1.08(1.01‒1.16)	0.028
**DM**	1.13(0.91‒1.40)	0.284		
**Medication**				
ACEI	1.15(0.83‒1.59)	0.408		
ARB	0.71(0.58‒0.88)	0.002	0.77(0.62‒0.96)	0.020
CCB	0.87(0.71‒1.06)	0.153		
α-blockers	1.04(0.76‒1.42)	0.814		
β-blockers	1.02(0.81‒1.27)	0.883		
Furosemide	1.68(1.33‒2.14)	< 0.001	1.42(1.10‒1.84)	0.008
Indapamide	0.21(0.07‒0.64)	0.006	0.21(0.07‒0.66)	0.007
Spironolactone	0.67(0.33‒1.36)	0.266		
Amiloride	0.62(0.33‒1.15)	0.128		
NSAIDs	1.20(0.97‒1.47)	0.084		
TCA	0.87(0.46‒1.61)	0.648		
Diazepam	1.05(0.71‒1.55)	0.806		

*Adjusted HRs were calculated after controlling for age, CCIS, DM, medications.

**Abbreviations: HR, hazard ratio; CI, confidence interval; TAH, thiazide-associated hyponatremia; CCIS, Charlson Comorbidity Index score; DM, diabetes mellitus; ACEI, angiotensin converting enzyme inhibitors; ARB, angiotensin II receptor blocker; CCB, calcium channel blocker; NSAIDs, non-steroidal anti-inflammatory drugs; TCA, tricyclic antidepressant.

**Fig. 2:**
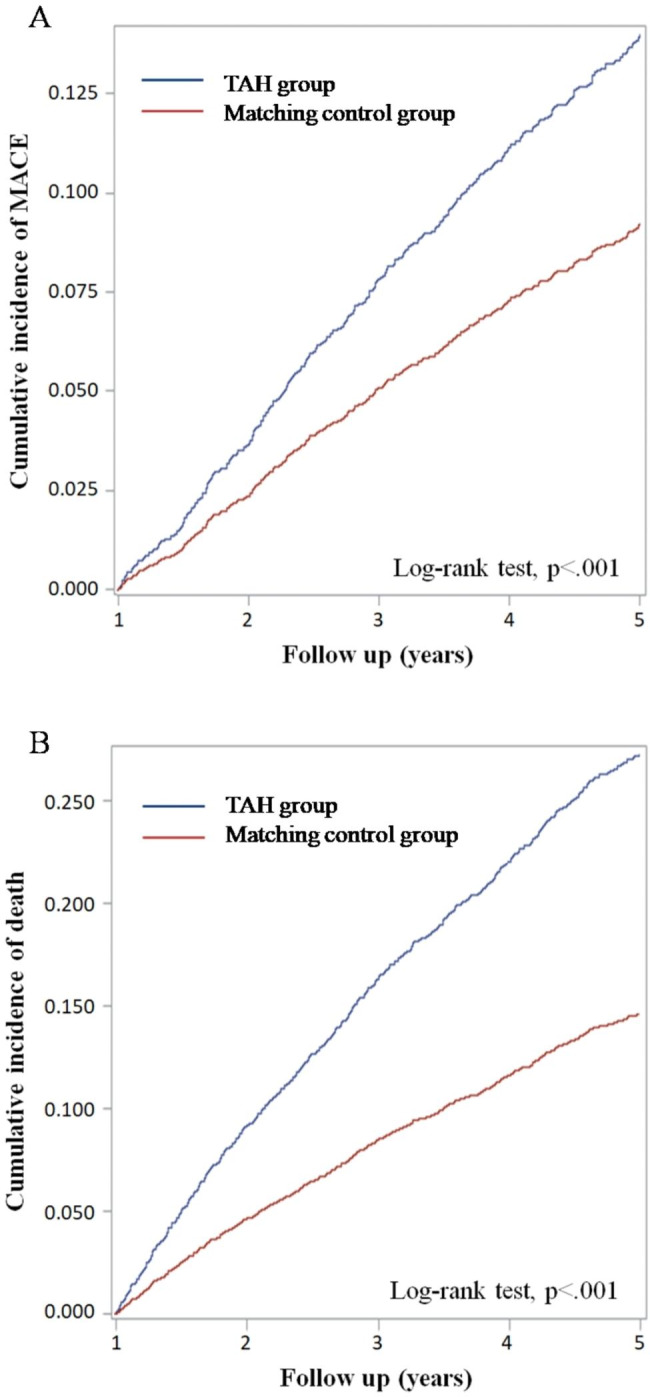
Kaplan-Meier cumulative survival curves for major cardiovascular events in the TAH group and control group

The Kaplan-Meier cumulative survival curves revealed significant differences in the incidence of MACE in the TAH group and control group (Fig. 2; *P* < 0.001, log rank test). The TAH group had a significantly higher cumulative rate of MACE (Fig. 2A) and all-cause death (Fig. 2B).

Further analysis of the incidence of MACE in the TAH group and control group is presented in Table 3. The incidence of MACE in the TAH group was 3.20 / 100 person-years and 1.95 / 100 person-years in the control group. The incidence of all-cause death in the TAH group was 6.24 / 100 person-years and 3.13 / 100 person-years in the control group. After expressing the incidence rate ratio (TAH group versus control group) as hazard ratio, the TAH group had a HR of 1.29 (CI 1.01‒1.65) for MACE, HR of 1.39 (CI 1.19‒1.63) for all-cause death, and HR of 1.61 (CI 1.14‒2.27) for stroke after adjusting for age, CCIS, DM, ARB, CCB, α-blockers, β-blockers, and furosemide.

**Table 3:** Incidence of MACE per 100 person-years in the TAH group and control group

	TAH group				Control group					
Event	No. of events	Person- years	Incidence rate		No. of Event	Person- years	Incidence rate	Crude HR* (95%CI)		Adjusted HR** (95%CI)
MACE	128	4001	3.20		254	13,025	1.95	1.56(1.26‒1.92)		1.29(1.01‒1.65)
All-cause death	316	5066	6.24		507	16,199	3.13	2.02(1.75‒2.32)		1.39(1.19‒1.63)
CV death	24	5066	0.47		36	16,199	0.22	2.01(1.20‒3.37)		1.61(0.90‒2.92)
Major coronary	65	4001	1.62		129	13,025	0.99	1.56(1.16‒2.11)		1.07(0.76‒1.50)
Stroke	63	4001	1.57		125	13,025	0.96	1.56(1.15‒2.11)		1.61(1.14‒2.27)

*Incidence rate ratio: TAH group versus control group (95%CI).

**Adjusted for age, CCIS, DM, ARB, CCB, α-blockers, β-blockers, and furosemide.

## Discussion

This study indicates that TAH adversely increases the risk of MACE among elderly individuals prescribed thiazide diuretics in Taiwan. Diagnosis of the complication thiazide hyponatremia was associated with a significantly increased risk of MACE in patients over 65-years-old: TAH was associated with 29% higher risk of MACE, 39% higher risk of all-cause death and 61% higher risk of stroke. These results remained statistically significant after adjustment for other known risk factors.

TAH occurs when an euvolemic patient is prescribed thiazide diuretics but then develops hyponatremia. The precise mechanisms underlying thiazide-induced hyponatremia are complex and have not been fully examined, and the onset time is unpredictable [24, 25]. A variety of processes, including excessive water intake, impaired free-water excretion, solute depletion, and osmotic inactivation of cations can occur in elderly patients [26]. Factors related to age may partially explain the uncertainty of the time to onset of TAH [27]. Moreover, females have been found to be at higher risk of TAH [8]. One potential explanation is that the thiazide-sensitive sodium chloride co-transporter (NCC) may be expressed at higher levels in females [28, 29]. Other sex-related factors such as higher ADH release [30] and gene expression of the prostaglandin transporter *SLCO2A1*, which reduces the activity of the PGE2 transporter [31], may play a role in the higher risk of TAH in females. In addition, the rs2509585 C/T or T/T polymorphisms in the *KCNJ1* (Potassium Channel, Inwardly Rectifying Subfamily J, Member 1) gene were recently associated with higher risk of severe TAH in older female patients in Taiwan [32].

The term “thiazide diuretics” encompasses all diuretics thought to have a major effect on the distal tubule. Although thiazide-type and thiazide-like diuretics differ in their drug structure, thiazide-like diuretics are sulfonamides that lack the molecular structure of benzothiadiazine. Though do not have the same chemical properties as thiazide-type diuretics, thiazide-like diuretics exert similar physiological effects as thiazide-type diuretics [33]. While some studies have suggested that they should be classified as different drugs, other studies have shown that all drugs in the thiazide class act equally effectively on the distal convoluted tubule [34]. However, numerous studies have suggested that the cardiovascular outcomes of hydrochlorothiazide and chlorthalidone are not necessarily the same due to their varied pharmacokinetic and pharmacodynamic features [35–41].

Based on the current hypertension guidelines of the JNC 8, thiazide diuretics are recommended as the initial drug for the general non-black population. This recommendation was supported by the results of the Antihypertensive and Lipid-Lowering Treatment to Prevent Heart Attack Trial (ALLHAT) [1] and Systolic Hypertension in the Elderly Program (SHEP) [2]. However, fewer than 60% of the patients in the ALLHAT study were 65 or older and patients with other ethnicities (such as Asian) represented less than 5% of participants [1]. Thus, the ALLHAT study may have not accurately reflected the risk of thiazide complications in the elderly population and has a general selection bias due to the low numbers of people of Eastern Asian ethnicity. In addition, the ALLHAT trial used chlorthalidone as the first-step thiazide diuretic. While the SHEP trial only studied patients older than 60-years-old, the results showed that low-dose chlorthalidone as step 1 medication reduced the risk of MACE [2]. These results conflict with the findings of this study. We only assessed patients prescribed thiazide-type diuretics instead of thiazide-like diuretics, which may probably explain the discrepancies between our study and the ALLHAT and SHEP trials.

Several recent articles have drawn attention to TAH in the elderly [9, 20, 26, 42, 43] and discussed the associated risk of cardiovascular events [18]. Therefore, we conducted this study to provide more conclusive evidence on the associations between TAH and the risk of MACE in elderly Taiwanese patients. This study provides several contributions. First, this was a population-based retrospective observational study of the risk of MACE among elderly patients with TAH in Taiwan. We assessed two million subjects; hence, the results of this study provide strong evidence of the higher risk of MACE among patients with the drug complication TAH. Second, the cardiovascular benefits of thiazide diuretics observed in previous trials may be due to good blood pressure control. However, our study highlights the side effects of thiazide diuretics and risks associated with TAH in the elderly. Third, the population of this study were all 65-years-oldor above, thus this work provides evidence for current practice and future research in geriatrics. Forth, thiazide conjugated anti-hypertension medications (such as Exforge HCT, Sevikar HCT, Co-Diovan, & Hyzaar) were also included in this study, which may remind us to monitor sodium level under insidious dose.

Despite these advantages, there are still several inevitable limitations to this study. First, laboratory data such as sodium levels and other clinical parameters could not be obtained from the national health insurance research database (NHIRD). We made efforts to establish an adequate study design to overcome this limitation, well-designed observational studies can yield valid outcomes. Analysis of a nationwide-scale database such as the NHIRD can provide relevant results. Second, no blood pressure data were available, thus we could not assess blood pressure control in the TAH and control groups. Third, information on self-paid medications and over-the-counter drugs is not available in the NHIRD and mild hyponatremia may not be recorded as a primary diagnosis. Our analysis could not overcome this essential defect, thus our results may underestimate the actual risk of MACE associated with TAH.

Hyponatremia is rare when taking SSRIs (selective serotonin reuptake inhibitors) and may be caused by an imbalance in the secretion of antidiuretic hormone (SIADH). We acknowledged that the lack of inclusion of SSRIs analysis was a design flaw. In our study design, we preferred to focus on direct drug effect of thiazide instead of the rare side effect of SSRIs. However, tricyclic antidepressants (TCA) are included in the medication analysis. The Table-1 medication block had listed TCA was 5.1% in TAH group while 2.1% in control group. Which may reflect the use of antidepressant agent seemed a little higher in TAH group. Taking with diazepam, it also showed the same trend. But in Cox model, both showed no significant HR.

The exact number of study group source (inpatient, outpatient, or ER) was not well distinguished, but it was large proportion from ER and inpatient under Taiwan’s medical settings. For this retrospective study, we only tried to connect the association of diagnosis and medication with MACE without further distinguishing the source of patients.

Given the assumption that other classes of drugs or combinations of drugs those lead to increased all-cause mortality, the difference of MACE in TAH and control group were still firm in our study. In Table 1, we have listed the common medication combined with thiazide and Table 2 presented multivariable Cox proportional hazard model for risk of MACE in each drug. The results were turbulence and no significant HR in most drugs.

Given the universal use of thiazide diuretics, we suggest this medication should be stopped once the complication of hyponatremia occurs, and the medication should be reviewed or shifted to another class of diuretic. Furthermore, thiazides should be prescribed with caution to older patients and these individuals should be monitored carefully for TAH. We hope that this study will encourage clinicians to be more cautious when prescribing thiazide diuretics as a treatment for hyponatremia in elderly patients in Taiwan.

## Conclusions

TAH increases the risk of MACE in the elderly in Taiwan. A diagnosis of TAH increases the risk of MACE by 29%, all-cause death by 39% and stroke by 61% in ≥ 65-year-olds.

### Electronic supplementary material

Below is the link to the electronic supplementary material.

Supplementary Material 1

## Data Availability

The data that support the findings of this study are available from National Health Insurance Research Database of Taiwan, but restrictions apply to the availability of these data, which were used under license for the current study, and so are not publicly available. Data are however available from the authors upon reasonable request and with permission of National Health Insurance Research Database of Taiwan (http://nhird.nhri.org.tw/en/index.htm).
