# Supplementary material for: Thiazide-associated hyponatremia increases the risk of major adverse cardiovascular events among elderly Taiwanese patients
Source: BMC Geriatr. 2023 Dec 15;23:862. doi: 10.1186/s12877-023-04583-w (PMC10724905; doi:10.1186/s12877-023-04583-w)
Supplement: Supplementary file 1 — Supplementary Material 1 [file 12877_2023_4583_MOESM1_ESM.docx]

Supplement – ATC code of medicines containing thiazides available in Taiwan from 2002 to 2012

| **ATC code** | **Drug Name** | **Composition** | **Pharmaceutical factory** |
| --- | --- | --- | --- |
| C02LA01 | HYTHIAPINE TABLETS | RESERPINE 100MCG + HYDROCHLOROTHIAZIDE 10MG | Bora Pharmaceuticals Ophthalmic Inc. |
| C02LA01 | BEHYD RA TABLETS | RESERPINE 100MCG + BENZYLHYDROCHLOROTHIAZIDE 4MG | Shinlin Sinseng Pharmaceutical Co.,Ltd |
| C02LA01 | H.R.P TABLETS | RESERPINE 125MCG + HYDROCHLOROTHIAZIDE 25MG | Souriree Biotech Pharmaceutical Company, Ltd. |
| C02LA01 | REDREL TSBLETS | RESERPINE 125MCG + HYDROCHLOROTHIAZIDE 25MG | Souriree Biotech Pharmaceutical Company, Ltd. |
| C02LA50 | ISO-TRIRAUPIN S.P. DRAGEES | BUTIZIDE=THIABUTAZIDE=ISOBUTYLHYDROCHO | HARVESTER TRADING CO., LTD. |
| C02LA51 | RELAPINE TABLETS "C.H." | RESERPINE 100MCG + HYDRALAZINE 10MG + HYDROCHLOROTHIAZIDE 10MG | CHEN HO PHARMACEUTICAL CO., LTD. |
| C02LA51 | R.H.H. TABLETS "JOHNSON" | RESERPINE 100MCG + HYDRALAZINE 10MG + HYDROCHLOROTHIAZIDE 10MG | Johnson Chemical Pharmaceutical Works CO.LTD |
| C02LA51 | TRIDEAL TABLETS "IDEAL" | RESERPINE 100MCG + HYDRALAZINE 10MG + HYDROCHLOROTHIAZIDE 10MG | LI HSIANG PHARMACEUTICAL CO., LTD. |
| C02LA51 | ESIDAR S.C. TABLETS "M.T." | RESERPINE 100MCG + HYDRALAZINE 10MG + HYDROCHLOROTHIAZIDE 10MG | MING TA CHEMISTRY PHARMACY CO., LTD. |
| C02LA51 | R.H.H. TABLETS "N.M." | RESERPINE 100MCG + HYDRALAZINE 25MG + HYDROCHLOROTHIAZIDE 15MG | NAN ME PHARMACEUTICAL PRODUCTION CO., LTD. |
| C02LA51 | TRIANPRES S.C. TABLETS "N.C.P." | RESERPINE 100MCG + HYDRALAZINE 10MG + HYDROCHLOROTHIAZIDE 10MG | NEW CHEMICAL & PHARMACEUTICAL LABORATORY CO., LTD. |
| C02LA51 | ESIDRI S.C. TABLETS (R) | RESERPINE 100MCG + HYDRALAZINE 10MG + HYDROCHLOROTHIAZIDE 10MG | Novartis (Taiwan) Co., Ltd. |
| C02LA51 | DEHYDRI S.C. TABLETS "P.J." | RESERPINE 100MCG + HYDRALAZINE 10MG + HYDROCHLOROTHIAZIDE 10MG | PEI JIN INTERNATIONAL CO., LTD. |
| C02LA51 | LISEIPIN F.C. TABLETS "ROOT" | RESERPINE 100MCG + HYDRALAZINE 25MG + HYDROCHLOROTHIAZIDE 15MG | ROOT CHEMICAL PHARMACY CO., LTD. |
| C02LA51 | TRISDOWN F.C TABLETS "ROYAL" | RESERPINE 100MCG + HYDRALAZINE 10MG + HYDROCHLOROTHIAZIDE 10MG | ROYAL CHEMICAL & PHARMACEUTICAL CO., LTD. |
| C02LA51 | RELAZIDE S.C. TABLETS "STANDARD" | RESERPINE 100MCG + HYDRALAZINE 10MG + HYDROCHLOROTHIAZIDE 10MG | Standard Chem & Pharm CO., LTD. |
| C02LA51 | SERPATHIAZIDE F.C. TABLETS "WST" | RESERPINE 100MCG + HYDRALAZINE 25MG + HYDROCHLOROTHIAZIDE 15MG | WASHINGTON PHARMACEUTICAL CO., LTD. |
| C02LA51 | SERAZIDE TABLETS "WINSTON" | RESERPINE 100MCG + HYDRALAZINE 25MG + HYDROCHLOROTHIAZIDE 15MG | WINSTON MEDICAL SUPPLY CO., LTD. |
| C02LA51 | R.H.H TABLETS "Y.Y" | RESERPINE 100MCG + HYDRALAZINE 10MG + HYDROCHLOROTHIAZIDE 10MG | YING YUAN CHEMICAL PHARMACEUTICAL CO., LTD. |
| C02LA51 | RESERZIDE S.C. TABLETS | RESERPINE 100MCG + HYDRALAZINE 10MG + HYDROCHLOROTHIAZIDE 10MG | YUAN CHOU CHEMICAL AND PHARMACEUTICAL CO., LTD. |
| C02LB01 | HYDROMET TABLETS | METHYLDOPA 250MG + HYDROCHLOROTHIAZIDE 15MG | Merck & Company, Inc. (MSD) |
| C02LG02 | ANPRELINE TABLETS "OEIENTAL" | HYDRALAZINE 25MG + HYDROCHLOROTHIAZIDE 15MG | ORIENTAL CHEMICAL WORKS INC. |
| C03AA | BEHYD TABLETS | BENZYLHYDROCHLOROTHIAZIDE , 4.00 MG | Shinlin Sinseng Pharmaceutical Co.,Ltd |
| C03AA03 | NISIDREX TABLETS | HYDROCHLOROTHIAZIDE , 25.00 MG | BEST YOUNG BIOTECH CO., LTD. |
| C03AA03 | DECAZON TABLETS "CBC" | HYDROCHLOROTHIAZIDE , 50.00 MG | CBC BIOTECHNOLOGICAL & PHARMACEUTICAL CO., LTD. |
| C03AA03 | HYDRO DIUL TABLETS | HYDROCHLOROTHIAZIDE , 25.00 MG | Center Laboratories, Inc. |
| C03AA03 | COLONRAITAI TABLETS "CHINTENG" | HYDROCHLOROTHIAZIDE , 25.00 MG | CHIN TENG INTERNATIONAL PHARMACEUTICAL MANUFACTURE CORP. |
| C03AA03 | DIHYDRODIAZID TABLETS | HYDROCHLOROTHIAZIDE , 25.00 MG | China Chemical & Pharmaceutical Co., Ltd. |
| C03AA03 | KESHIAU TABLETS 25MG "F.S." | HYDROCHLOROTHIAZIDE , 25.00 MG | FUSENG PHARMACEUTICAL FACTORY CO., LTD. |
| C03AA03 | HYDROCHLOROTHIAZIDE TABLETS "HONTEN" | HYDROCHLOROTHIAZIDE , 50.00 MG | HON TEN PHARMACERUTICAL CO., LTD. |
| C03AA03 | HYDROCHLOROTHIAZIDE TABLETS 25MG "JEN SHENG" | HYDROCHLOROTHIAZIDE , 25.00 MG | Jen Sheng Pharmaceutical Co., Ltd |
| C03AA03 | HYCHLOZIDE TABLETS | HYDROCHLOROTHIAZIDE , 25.00 MG | Johnson Chemical Pharmaceutical Works CO.LTD |
| C03AA03 | DICHLOTRIDE TABLETS 25MG | HYDROCHLOROTHIAZIDE , 25.00 MG | Merck & Company, Inc. (MSD) |
| C03AA03 | DICHLOTRIDE TABLETS 50MG | HYDROCHLOROTHIAZIDE , 50.00 MG | Merck & Company, Inc. (MSD) |
| C03AA03 | HYDROCHLOROTHIAZIDE TABLETS "N.C.P." | HYDROCHLOROTHIAZIDE , 25.00 MG | NEW CHEMICAL & PHARMACEUTICAL LABORATORY CO., LTD. |
| C03AA03 | ESIDREX TABLETS | HYDROCHLOROTHIAZIDE , 25.00 MG | Novartis (Taiwan) Co., Ltd. |
| C03AA03 | DIHYDROCHLOROTHIAZIDE TABLETS "ORIENTAL" | HYDROCHLOROTHIAZIDE , 25.00 MG | ORIENTAL CHEMICAL WORKS INC. |
| C03AA03 | HYDROCHLOROTHIAZIDE TABLETS "SHINLON" | HYDROCHLOROTHIAZIDE , 25.00 MG | Shinlon Pharmaceutical Ind. co., LTD |
| C03AA03 | DICOMTRIDE TABLETS 50MG (HYDROCHLOROTHIAZIDE)"COMFORT" | HYDROCHLOROTHIAZIDE , 50.00 MG | Souriree Biotech Pharmaceutical Company, Ltd. |
| C03AA03 | DIHYDROCHL OZIDE TABLETS. | HYDROCHLOROTHIAZIDE , 25.00 MG | Tah-An Chemical and Pharmaceutical Co., Ltd. |
| C03AA03 | HYDROCHLOROTHIAZIDE TABLETS "VPP" | HYDROCHLOROTHIAZIDE , 50.00 MG | TAIWAN VETERANS PHARMACEUTICAL CO., LTD. |
| C03AA03 | CHUNCOTOL TABLETS "Y.S." | HYDROCHLOROTHIAZIDE , 25.00 MG | Taiwan Yang Sheng Pharmaceutical Ind. Co., Ltd. |
| C03AA03 | HYBOZIDE TAB 50MG | HYDROCHLOROTHIAZIDE , 50.00 MG | Tai-Yu chemical and pharmaceutical co., Ltd. |
| C03AA03 | KOLISIDE TABLETS 25MG | HYDROCHLOROTHIAZIDE , 25.00 MG | TEN SHARP BIOTECH CO., LTD. |
| C03AA03 | DIHYDROCHLOROTHIAZIDE TABLETS | HYDROCHLOROTHIAZIDE , 25.00 MG | Update Pharmaceutical Co.﹐LTD. |
| C03AA03 | DITHIAZIDE TABLETS | HYDROCHLOROTHIAZIDE , 25.00 MG | WASHINGTON PHARMACEUTICAL CO., LTD. |
| C03AA03 | LISUZONE TABLETS "Y.K."(HYDROCHLOROTHIAZIDE) | HYDROCHLOROTHIAZIDE , 25.00 MG | York Pharmaceutical Co., Ltd. |
| C03AA06 | DOQUA TABLETS (TRICHLORMETHIAZIDE) | TRICHLORMETHIAZIDE , 4.00 MG | HCT PHARMA WORKS CO., LTD. |
| C03AA06 | TRICOZIDE TABLETS "JOHNSON" | TRICHLORMETHIAZIDE , 2.00 MG | Johnson Chemical Pharmaceutical Works CO.LTD |
| C03AA06 | ILIYA TABLETS (TRICHLORMETHIAZIDE)"CURIE" | TRICHLORMETHIAZIDE , 2.00 MG | Panion & BF Biotech Inc. |
| C03AA06 | WUPIN TABLETS "P.L." | TRICHLORMETHIAZIDE , 4.00 MG | PEI LI PHARMACEUTICAL INDUSTRIAL CO., LTD. |
| C03AA06 | EAZIDE TABLETS | TRICHLORMETHIAZIDE , 2.00 MG | SWISS PHARMACEUTICAL CO., LTD. |
| C03AA06 | TRIKLOR TABLETS 2MG | TRICHLORMETHIAZIDE , 2.00 MG | SYNRAY BIOTECH COMPANY LIMITED |
| C03AA06 | FLUITRAN TABLETS | TRICHLORMETHIAZIDE , 2.00 MG | Taiwan Shionogi & Co., Ltd. |
| C03AA06 | FLUITRAN TABLETS | TRICHLORMETHIAZIDE , 2.00 MG | Taiwan Shionogi & Co., Ltd. |
| C03AA07 | NAVIDREX TABLETS 0.25MG (CYCLOPENTHIAZIDE) | CYCLOPENTHIAZIDE , 250.00 MCG | Novartis (Taiwan) Co., Ltd. |
| C03EA | ALDACTIDE 25 | HYDROFLUMETHIAZIDE 25MG + SPIRONOLACTONE 25MG | PFIZER LIMITED |
| C03EA01 | MAXURINE TABLET "KINGDOM" | HYDROCHLOROTHIAZIDE 50MG + TRIAMTERENE 75MG | Bora Pharmaceuticals Ophthalmic Inc. |
| C03EA01 | TIADEN TABLETS | AMILORIDE 5MG + HYDROCHLOROTHIAZIDE 50MG | CENTAPHARM INC. |
| C03EA01 | ANZA TABLETS "CHINTENG" | AMILORIDE 5MG + HYDROCHLOROTHIAZIDE 50MG | CHIN TENG INTERNATIONAL PHARMACEUTICAL MANUFACTURE CORP. |
| C03EA01 | DEPRESS TABLETS "CHINTENG" | HYDROCHLOROTHIAZIDE 25MG + TRIAMTERENE 50MG | CHIN TENG INTERNATIONAL PHARMACEUTICAL MANUFACTURE CORP. |
| C03EA01 | SPILAZIDE TABLETS "C.R." | HYDROCHLOROTHIAZIDE 25MG + SPIRONOLACTONE 25MG | CHURYO MEDICAL CORPORATION |
| C03EA01 | DIUREN TABLETS "GCPC" | HYDROCHLOROTHIAZIDE 25MG + TRIAMTERENE 50MG | GENUINE CHEMICAL PHARMACEUTICAL CO., LTD. |
| C03EA01 | DYAZIDE TABLETS | HYDROCHLOROTHIAZIDE 25MG + TRIAMTERENE 50MG | GLAXOSMITHKLINE FAR EAST B.V. |
| C03EA01 | DAZID TABLETS "H.S" | HYDROCHLOROTHIAZIDE 25MG + TRIAMTERENE 50MG | Huashin Chemical Pharmaceutical Works Co., Ltd. |
| C03EA01 | RIYAZINE CAPSULES "H.S." | HYDROCHLOROTHIAZIDE 25MG + TRIAMTERENE 50MG | Huashin Chemical Pharmaceutical Works Co., Ltd. |
| C03EA01 | AMITON TABLETS "H.S." | AMILORIDE 5MG + HYDROCHLOROTHIAZIDE 50MG | Hwangs Pharmaceutical co., Ltd. |
| C03EA01 | EDEPRESS CAPSULES "SHIN FONG" | HYDROCHLOROTHIAZIDE 25MG + TRIAMTERENE 50MG | JENN MAW CO., LTD. |
| C03EA01 | MAXZIDE TABLETS "MEIDER" | HYDROCHLOROTHIAZIDE 25MG + TRIAMTERENE 50MG | MEIDER PHARMACEUTICAL CO., LTD. |
| C03EA01 | MODURETIC TABLETS | AMILORIDE 5MG + HYDROCHLOROTHIAZIDE 50MG | Merck & Company, Inc. (MSD) |
| C03EA01 | MAXPRESS TABLET | HYDROCHLOROTHIAZIDE 25MG + TRIAMTERENE 50MG | SIU GUAN CHEMICAL INDUSTRIAL CO., LTD. |
| C03EA01 | AMIZIDE TABLETS "STANDARD" | AMILORIDE 5MG + HYDROCHLOROTHIAZIDE 50MG | Standard Chem & Pharm CO., LTD. |
| C03EA01 | AMILCO TABLETS | AMILORIDE 5MG + HYDROCHLOROTHIAZIDE 50MG | SUPER FORTUNE ENTERPRISE CO., LTD. |
| C03EA01 | EDENIL TABLETS | AMILORIDE 5MG + HYDROCHLOROTHIAZIDE 50MG | SWISS PHARMACEUTICAL CO., LTD. |
| C03EA01 | AMITRID TABLETS | AMILORIDE 5MG + HYDROCHLOROTHIAZIDE 50MG | TRIFO LIMITED |
| C03EA01 | DIURET CAPSULES | HYDROCHLOROTHIAZIDE 25MG + TRIAMTERENE 50MG | TTY BIOPHARM COMPANY LIMITED |
| C03EA01 | SLOSAT TABLETS "WEIDAR" | HYDROCHLOROTHIAZIDE 25MG + SPIRONOLACTONE 25MG | WEIDAR CHEMICAL & PHARMACEUTICAL CO., LTD. |
| C03EA01 | TRIAMZIDE CAPSULES "WEIDAR" | HYDROCHLOROTHIAZIDE 25MG + TRIAMTERENE 50MG | WEIDAR CHEMICAL & PHARMACEUTICAL CO., LTD. |
| C03EA01 | URINIS CAPSULES "YY" | HYDROCHLOROTHIAZIDE 25MG + TRIAMTERENE 50MG | YING YUAN CHEMICAL PHARMACEUTICAL CO., LTD. |
| C03EA01 | ANJAL TABLETS "YU SHENG" | HYDROCHLOROTHIAZIDE 25MG + TRIAMTERENE 50MG | YU SHENG PHARMACEUTICAL CO., LTD. |
| C03EA01 | ALOZIDE TABLETS | AMILORIDE 5MG + HYDROCHLOROTHIAZIDE 50MG | YUAN CHOU CHEMICAL AND PHARMACEUTICAL CO., LTD. |
| C07BA68 | TORRALIS TABLETS | METIPRANOLOL 20MG + BUTHIAZIDE 2.5MG | HARVESTER TRADING CO., LTD. |
| C07BB02 | BETAZIDE TABLETS | METOPROLOL 100MG + HYDROCHLOROTHIAZIDE 12.5MG | AstraZeneca |
| C07DA06 | MODUCREN TABLETS | TIMOLOL 10MG + AMILORIDE 2.5MG + HYDROCHLOROTHIAZIDE 25MG | Merck & Company, Inc. (MSD) |
| C09BA01 | ZUZIDE TAB. | CAPTOPRIL 25MG + HYDROCHLOROTHIAZIDE 15MG | CHIN TENG INTERNATIONAL PHARMACEUTICAL MANUFACTURE CORP. |
| C09BA01 | LOWERNIN TABLETS "H.S." | CAPTOPRIL 25MG + HYDROCHLOROTHIAZIDE 15MG | Huashin Chemical Pharmaceutical Works Co., Ltd. |
| C09BA02 | LANDUET TABLETS 10MG/25MG | ENALAPRIL 10MG +HYDROCHLOROTHIAZIDE 25MG | YUNG SHIN PHARM. IND. CO., LTD. |
| C09BA02 | LANDUET TABLETS 5MG/12.5MG | ENALAPRIL 5MG +HYDROCHLOROTHIAZIDE 12.5MG | YUNG SHIN PHARM. IND. CO., LTD. |
| C09DA01 | FAXIVEN FILM COATED TABLET (50+12.5) MG | LOSARTAN 50MG + HYDROCHLOROTHIAZIDE 0-12.5MG | AKTIVE ENTERPRISE CO., LTD. |
| C09DA01 | Candanxo F.C. Tablets 50/12.5 mg | LOSARTAN 50MG + HYDROCHLOROTHIAZIDE 0-12.5MG | Anxo Pharmaceutical Co., Ltd |
| C09DA01 | ZOSAAHY F.C TABLETS 50/12.5MG | LOSARTAN 50MG + HYDROCHLOROTHIAZIDE 0-12.5MG | China Chemical & Pharmaceutical Co., Ltd. |
| C09DA01 | LOSA&HYDRO F.C. TABLETS 50/12.5MG "CYH" | LOSARTAN 50MG + HYDROCHLOROTHIAZIDE 0-12.5MG | CHUNGHWA YUMING HEALTHCARE CO., LTD. |
| C09DA01 | SYNZAR F.C. TABLETS 50/12.5 MG | LOSARTAN 50MG + HYDROCHLOROTHIAZIDE 0-12.5MG | DIN FENG YU BIOPHARM CO., LTD. |
| C09DA01 | HYZAAR FC TABLETS 100/12.5 MG | LOSARTAN 100MG + HYDROCHLOROTHIAZIDE 0-12.5MG | Merck & Company, Inc. (MSD) |
| C09DA01 | HYZAAR F.C TAB. 50/12.5MG | LOSARTAN 50MG + HYDROCHLOROTHIAZIDE 0-12.5MG | Merck & Company, Inc. (MSD) |
| C09DA01 | Losartan Sandoz Comp Film Coated Tablet 50/12.5 mg | LOSARTAN 50MG + HYDROCHLOROTHIAZIDE 0-12.5MG | Novartis (Taiwan) Co., Ltd.) |
| C09DA01 | HISART F.C. TABLETS 50/12.5MG"STANDARD"(LOSARTAN POTASSIUM AND HYDROCHLOROTHIAZIDE) | LOSARTAN 50MG + HYDROCHLOROTHIAZIDE 0-12.5MG | Standard Chem & Pharm CO., LTD. |
| C09DA01 | FUZATAN F.C. TABLETS 50/12.5MG "KINGDOM" | LOSARTAN 50MG + HYDROCHLOROTHIAZIDE 0-12.5MG, | SYNMOSA BIOPHARMA CORPORATION |
| C09DA01 | LOSARZIDE F.C. TABLETS 50/12.5MG | LOSARTAN 50MG + HYDROCHLOROTHIAZIDE 0-12.5MG | TAIWAN VETERANS PHARMACEUTICAL CO., LTD. |
| C09DA01 | LOSACAR-H TABLETS | LOSARTAN 50MG + HYDROCHLOROTHIAZIDE 0-12.5MG | Yi You Biomedical Co., Ltd. |
| C09DA03 | CO-TAREG FILM COATED TABLETS 160/12.5MG | VALSARTAN 160MG + HYDROCHLOROTHIAZIDE 0-25MG | ALCON SERVICES AG, TAIWAN BRANCH (SWITZERLAND) |
| C09DA03 | CO-TAREG FILM COATED TABLETS 80/12.5MG | VALSARTAN 80MG + HYDROCHLOROTHIAZIDE 0-12.5MG | ALCON SERVICES AG, TAIWAN BRANCH (SWITZERLAND) |
| C09DA03 | CO-DAIWEN CAPSULES 80/12.5MG | VALSARTAN 80MG + HYDROCHLOROTHIAZIDE 0-12.5MG | Anxo Pharmaceutical Co., Ltd |
| C09DA03 | CO-VOSAA FILM COATED TABLETS 80/12.5MG | VALSARTAN 80MG + HYDROCHLOROTHIAZIDE 0-12.5MG | China Chemical & Pharmaceutical Co., Ltd. |
| C09DA03 | COSARTAN 80/12.5 FILM COATED TABLETS "MACRO" | VALSARTAN 80MG + HYDROCHLOROTHIAZIDE 0-12.5MG | MACRO GLOBAL CORPORATION |
| C09DA03 | KOVAN PLUS Film Coated Tablets 80/12.5mg 〝MACRO〞 | VALSARTAN 80MG + HYDROCHLOROTHIAZIDE 0-12.5MG | MACRO GLOBAL CORPORATION |
| C09DA03 | CO-DIOVAN 160/12.5 FILM COATED TABLETS | VALSARTAN 160MG + HYDROCHLOROTHIAZIDE 0-25MG | Novartis (Taiwan) Co., Ltd. |
| C09DA03 | CO-DIOVAN 160/25 FILM-COATED TABLETS | VALSARTAN 160MG + HYDROCHLOROTHIAZIDE 0-25MG | Novartis (Taiwan) Co., Ltd. |
| C09DA03 | Co-Diovan 320/25 Film-Coated Tablet | VALSARTAN 320MG + HYDROCHLOROTHIAZIDE 0-25MG | Novartis (Taiwan) Co., Ltd.) |
| C09DA03 | Co-Diovan 320/12.5 Film-Coated Tablet | VALSARTAN 320MG + HYDROCHLOROTHIAZIDE 0-25MG | Novartis (Taiwan) Co., Ltd.) |
| C09DA03 | CO-DIOVAN 80/12.5 FILM COATED TABLETS | VALSARTAN 80MG + HYDROCHLOROTHIAZIDE 0-12.5MG | Novartis (Taiwan) Co., Ltd.) |
| C09DA03 | HYCOPRESS F.C. TABLETS 160/12.5MG | VALSARTAN 160MG + HYDROCHLOROTHIAZIDE 0-25MG | Standard Chem & Pharm CO., LTD. |
| C09DA03 | HYCOPRESS F.C. TABLETS 80/12.5MG | VALSARTAN 80MG + HYDROCHLOROTHIAZIDE 0-12.5MG | Standard Chem & Pharm CO., LTD. |
| C09DA04 | Co-Alvoprel 300mg/25mg film-coated tablets | IRBESARTAN 300MG + HYDROCHLOROTHIAZIDE 0-25MG | LOTUS PHARMACEUTICAL CO., LTD. |
| C09DA04 | IRBESARTAN 150MG+HYDROCHLOROTHIAZIDE 12.5MG SANDOZ FILM COATED TABLET | IRBESARTAN 150MG + HYDROCHLOROTHIAZIDE 0-12.5MG | Novartis (Taiwan) Co., Ltd. |
| C09DA04 | Irbesartan 300mg+Hydrochlorothiazide 25mg Sandoz Film Coated Tablet | IRBESARTAN 300MG + HYDROCHLOROTHIAZIDE 0-25MG | Novartis (Taiwan) Co., Ltd.) |
| C09DA04 | Irbesartan 300mg+Hydrochlorothiazide 12.5mg Sandoz Film Coated Tablet | IRBESARTAN 300MG + HYDROCHLOROTHIAZIDE 0-25MG | Novartis (Taiwan) Co., Ltd.) |
| C09DA04 | COAPROVEL 150MG/12.5MG FILM-COATED TABLETS | IRBESARTAN 150MG + HYDROCHLOROTHIAZIDE 0-12.5MG | Sanofi Taiwan Co., Ltd |
| C09DA04 | COAPROVEL 300MG/12.5MG FILM-COATED TABLES | IRBESARTAN 300MG + HYDROCHLOROTHIAZIDE 0-25MG | Sanofi Taiwan Co., Ltd |
| C09DA04 | COAPROVEL FILM-COATED TABLETS 300MG/25MG | IRBESARTAN 300MG + HYDROCHLOROTHIAZIDE 0-25MG | Sanofi Taiwan Co., Ltd |
| C09DA06 | CANDESARTAN AND HYDROCHLOROTHIAZIDE MYLAN 16MG/12.5MG | CANDESARTAN 16MG + HYDROCHLOROTHIAZIDE 0-12.5MG | MYLAN (TAIWAN) LIMITED |
| C09DA06 | BLOPRESS 16MG PLUS 12.5MG TABLETS | CANDESARTAN 16MG + HYDROCHLOROTHIAZIDE 0-12.5MG | Takeda Pharmaceuticals Taiwan,Ltd. |
| C09DA06 | BLOPRESS 8MG PLUS 12.5MG TABLETS | CANDESARTAN 8MG + HYDROCHLOROTHIAZIDE 0-12.5MG | Takeda Pharmaceuticals Taiwan,Ltd. |
| C09DA07 | MICARDIS PLUS TABLETS 40/12.5MG | TELMISARTAN 40MG + AMLODIPINE 0-10MG + HYDROCHLOROTHIAZIDE 0-12.5MG | Boehringer Ingelheim Taiwan Ltd. |
| C09DA07 | MICARDIS PLUS TABLETS 80/12.5 MG | TELMISARTAN 80MG + AMLODIPINE 0-10MG + HYDROCHLOROTHIAZIDE 0-25MG | Boehringer Ingelheim Taiwan Ltd. |
| C09DA07 | TELMISARTAN/HYDROCHLOROTHIAZIDE MYLAN 80MG/12.5MG | TELMISARTAN 80MG + AMLODIPINE 0-10MG + HYDROCHLOROTHIAZIDE 0-25MG | MYLAN (TAIWAN) LIMITED |
| C09DA07 | TELCARD H 80/25 | TELMISARTAN 80MG + AMLODIPINE 0-10MG + HYDROCHLOROTHIAZIDE 0-25MG | Yi You Biomedical Co., Ltd. |
| C09DA08 | OLMETEC PLUS 20/25MG, FILM COATED TABLETS | OLMESARTAN 20MG + HYDROCHLOROTHIAZIDE 0-25MG | DAIICHI SANKYO TAIWAN LTD. |
| C09DX01 | DAFIRO HCT 10/160/12.5MG FILM-COATED TABLETS | VALSARTAN 160MG + AMLODIPINE 10MG + HYDROCHLOROTHIAZIDE 0-25MG | ALCON SERVICES AG, TAIWAN BRANCH (SWITZERLAND) |
| C09DX01 | DAFIRO HCT 10/160/25MG FILM-CATED TABLETS | VALSARTAN 160MG + AMLODIPINE 10MG + HYDROCHLOROTHIAZIDE 0-25MG | ALCON SERVICES AG, TAIWAN BRANCH (SWITZERLAND) |
| C09DX01 | DAFIRO HCT 5/160/12.5MG FILM-COATED TABLETS | VALSARTAN 160MG + AMLODIPINE 5MG + HYDROCHLOROTHIAZIDE 0-25MG | ALCON SERVICES AG, TAIWAN BRANCH (SWITZERLAND) |
| C09DX01 | DAFIRO HCT 5/160/25MG FILM-COATED TABLETS | VALSARTAN 160MG + AMLODIPINE 5MG + HYDROCHLOROTHIAZIDE 0-25MG | ALCON SERVICES AG, TAIWAN BRANCH (SWITZERLAND) |
| C09DX01 | Exforge HCT Film Coated Tablets 10/160/12.5 mg | VALSARTAN 160MG + AMLODIPINE 10MG + HYDROCHLOROTHIAZIDE 0-25MG | Novartis (Taiwan) Co., Ltd.) |
| C09DX01 | Exforge HCT Film Coated Tablets 10/160/25 mg | VALSARTAN 160MG + AMLODIPINE 10MG + HYDROCHLOROTHIAZIDE 0-25MG | Novartis (Taiwan) Co., Ltd.) |
| C09DX01 | Exforge HCT Film Coated Tablets 5/160/25 mg | VALSARTAN 160MG + AMLODIPINE 5MG + HYDROCHLOROTHIAZIDE 0-25MG | Novartis (Taiwan) Co., Ltd.) |
| C09DX01 | Exforge HCT Film Coated Tablets 5/160/12.5 mg | VALSARTAN 160MG + AMLODIPINE 5MG + HYDROCHLOROTHIAZIDE 0-25MG | Novartis (Taiwan) Co., Ltd.) |
| C09DX03 | SEVIKAR HCT 20/5/12.5MG | OLMESARTAN 20MG + AMLODIPINE 5MG + HYDROCHLOROTHIAZIDE 0-12.5MG | DAIICHI SANKYO TAIWAN LTD. |
| C09DX03 | SEVIKAR HCT 40/10/12.5MG | OLMESARTAN 40MG + AMLODIPINE 10MG + HYDROCHLOROTHIAZIDE 0-25MG | DAIICHI SANKYO TAIWAN LTD. |
| C09DX03 | SEVIKAR HCT 40/10/25MG | OLMESARTAN 40MG + AMLODIPINE 10MG + HYDROCHLOROTHIAZIDE 0-25MG | DAIICHI SANKYO TAIWAN LTD. |
| C09DX03 | SEVIKAR HCT 40/5/25MG | OLMESARTAN 40MG + AMLODIPINE 5MG + HYDROCHLOROTHIAZIDE 0-25MG | DAIICHI SANKYO TAIWAN LTD. |
| C09DX03 | SEVIKAR HCT 40/5/12.5MG | OLMESARTAN 40MG + AMLODIPINE 5MG + HYDROCHLOROTHIAZIDE 0-25MG | DAIICHI SANKYO TAIWAN LTD. |
| C09DX03 | SEVIKAR HCT 40/5/25MG | OLMESARTAN 40MG + AMLODIPINE 5MG + HYDROCHLOROTHIAZIDE 0-25MG | DAIICHI SANKYO TAIWAN LTD. |
